# Awareness of alcohol marketing one year after initial implementation of Ireland’s Public Health (Alcohol) Act and during the COVID-19 pandemic

**DOI:** 10.1093/pubmed/fdab353

**Published:** 2021-10-09

**Authors:** Nathan Critchlow, Crawford Moodie

**Affiliations:** Institute for Social Marketing and Health, Faculty of Health Sciences and Sport, University of Stirling, Stirling FK9 4LA, Scotland; Institute for Social Marketing and Health, Faculty of Health Sciences and Sport, University of Stirling, Stirling FK9 4LA, Scotland

**Keywords:** Alcohol advertising regulation, Alcohol control, Alcohol marketing regulation, Ireland

## Abstract

**Background:**

The Republic of Ireland is introducing new controls on alcohol marketing, starting in November 2019 with restrictions on some outdoor and cinema advertising, and a ban on public transport advertising. We examined changes in marketing awareness one year after initial implementation and during the COVID-19 pandemic.

**Methods:**

Repeat online cross-sectional surveys with adults in Ireland conducted October 2019 (*n* = 1,007) and October 2020 (*n* = 1,020). Participants self-reported past-month awareness of alcohol marketing and completed the Alcohol Use Disorders Identification Test-Concise (AUDIT-C). Current drinkers were categorised as those reporting heavy episodic drinking at least monthly and higher-risk drinkers (≥5 AUDIT-C).

**Results:**

In both waves, most participants recalled some marketing awareness (94.1% vs. 93.8%). For 9/13 activities measured in both waves, there were decreases in the proportion reporting any awareness and frequency of awareness, including for the newly restricted activities. For example, any awareness of public transport advertising decreased between waves (*OR_Adj_* = 0.66, 95%CI: 0.53–0.81). In both waves, higher past-month awareness was associated with at least monthly heavy episodic drinking and higher-risk consumption.

**Conclusion:**

We recommend a precautionary interpretation. It is plausible that both Ireland’s initial controls and COVID-19 restrictions contributed to decreases in awareness, but longer-term evaluation is required to determine relative contribution.

## Introduction

In the Republic of Ireland (‘Ireland’), per-capita alcohol consumption is greater than across the European region.^1^ Furthermore, over a third of drinkers report Heavy Episodic Drinking (HED; ≥ 60 grams of pure alcohol) on a typical drinking occasion, with doing so more likely among younger adults, males, and more disadvantaged areas.^2^ Such consumption is associated with many individual and social harms and places a large burden on Ireland’s economy.^3^ A study of 21 European countries found that Ireland was one of only two where alcohol consumption had not declined during the early stages of the COVID-19 pandemic.^4^ Furthermore, despite restrictions on socialising and the forced closure of many on-trade premises, among drinkers in Ireland who reported that their alcohol use had changed during this period, more reported an increase in consumption than a decrease.^5^

Ireland have introduced the Public Health (Alcohol) Act (hereafter ‘the Act’) to reduce population consumption and concomitant harms. The Act contains measures including minimum pricing, mandatory product labelling, price promotion restrictions, and structural separation in some licensed premises (i.e., physical barrier separating alcohol from other products).^6–9^ The Act became law in October 2018 and components will be phased in by the incumbent Minister for Health. The Act also includes restrictions on marketing. From 12^th^ November 2019, alcohol advertising is prohibited outdoor near youth-orientated environments (unless part of licensed or production premises), on public transport or at transport hubs, or at the cinema (unless the film has an 18+ classification or the advertising is part of licenced premises in the cinema). Children’s clothing that promotes alcohol is also prohibited. From 12^th^ November 2021, there will also be a prohibition on advertising in (or on) a sporting area during a sporting event and sponsorship of events aimed at children (or where most participants are children) or involving motor vehicles. Plans to place time restrictions on radio and television advertising, restrict print publication advertising, and limit advertising to factual information, do not have implementation dates.

There is a wealth of evidence, including longitudinal data, that exposure to alcohol marketing (including self-reported awareness) is causally linked to consumption, including higher-risk drinking.^10–13^ Research has also demonstrated how alcohol marketing influences a network of underlying psychological processes that are antecedent to consumption, including expectancies, norms, and brand salience.^14–17^ To date, however, most research has focused on consumers under the minimum legal purchasing age. There is comparatively less understanding about the reach and impact of marketing on adults, including vulnerable groups such as dependent drinkers, despite being primary and legal targets for marketing.^18^^,^^19^

The World Health Organization (WHO) recommend statutory controls on alcohol advertising as one of their ‘best buys’ to address alcohol-related harms^20^ and several countries already have such legislation.^21–24^ There is some content analysis research which has examined changes in marketing activity pre-and-post-restrictions^25^^,^^26^ and econometric studies that have examined the impact of advertising bans on consumption, albeit the latter are often dated, inconclusive, or are subject to methodological limitations.^27^ To date, however, there is a lack of consumer research examining the real-world impact that statutory controls have on marketing awareness and the association with consumption.^28^ This contrasts with consumer evaluations of marketing restrictions for other fast-moving consumer goods, such as tobacco^29–31^ and elsewhere for alcohol (e.g., minimum unit pricing or warning labels).^32^^,^^33^ Comparable consumer data is important nationally, to evaluate whether Ireland’s controls achieve their goals, and internationally, to inform debates about introducing similar restrictions elsewhere.

This study examines changes in alcohol marketing awareness one-year after implementation of the Act’s initial advertising controls: outdoor, public transport, cinema, and branded children’s clothing. Our sample is the under-researched population of adult consumers,^18^^,^^19^ which allows us to examine whether marketing is associated with risky consumption among legal drinkers.^34^ The second wave of data also provides insight into how restrictions on social interactions in response to COVID-19 have influenced marketing awareness. This complements research examining how alcohol companies adapted marketing practices during the pandemic^35^^,^^36^ and will provide insight into the possible contribution of marketing towards levels of consumption in Ireland during this period.^4^^,^^5^

## Methods

### Design and COVID-19 context

The data come from the first two waves of a repeat cross-sectional survey exploring the impact of the Act’s marketing restrictions. The first wave was conducted 14-25^th^ October 2019, the month before the initial restrictions were implemented.^6–9^ The second wave was conducted 8-18^th^ October 2020, during the COVID-19 pandemic. During the second wave, Ireland operated a ‘levels’ approach to pandemic restrictions.^37^ These ranged from level one (most lenient, e.g., some social contact permitted and some on-trade venues open with protective measures) to level five (most restrictive, e.g., stay at home); a full summary of the restrictions is provided in Supplementary Table 1. In the month prior to data collection—the timeframe given for self-reporting marketing awareness—most regions were in level two or three. Closer to data collection, it was recommended that all regions move to level five.^38^ The Government rejected this recommendation on 5^th^ October 2020,^39^ but all regions were moved to level three, which included a ban on indoor dining in hospitality premises and closure of cultural venues. A decision for all regions to enter level five came the day after data collection ended,^40^ indicating that pandemic severity and restrictions were escalating during data collection.

**Table 1:** Sample characteristics and alcohol consumption by survey wave

	W1 (October 2019: Pre-restrictions, pre-COVID-19)				W2 (October 2020: Post-restrictions, COVID-19)			
	Unweighted		Weighted		Unweighted		Weighted	
Variable	%	n	%	n	%	n	%	n
**Gender**								
Male	44.5	448	49.6	499	49.8	508	49.6	506
Female	55.5	559	50.4	508	50.2	512	50.4	514
**Age group**								
18–24 years	12.1	122	11.0	111	11.9	121	11.1	113
25–34 years	18.4	185	17.1	172	18.4	188	16.6	169
35–44 years	22.6	228	21.5	217	22.1	225	21.3	217
45–54 years	20.1	202	18.1	182	19.6	200	18.3	187
55+ years	26.8	270	32.3	325	28.0	286	32.7	334
**Region/County**								
Dublin	30.9	311	28.3	285	33.3	340	28.3	289
Rest of Leinster	26.3	265	27.0	272	25.0	255	27.0	275
Munster	24.3	245	26.9	271	24.4	249	26.9	274
Connaught & part of Ulster^a^	18.5	186	17.8	179	17.3	176	17.8	182
**Drinking status**								
Non-drinker	10.4	102	10.5	103	10.7	107	10.9	109
Current drinker	89.6	883	89.5	882	89.3	891	89.1	889
Not stated	-	22	-	22	-	22	-	22
**Heavy Episodic Drinking (HED)** ^**b**^								
Never/Less than monthly	56.6	489	56.2	486	56.5	494	57.2	499
At least monthly	43.4	375	43.8	378	43.5	381	42.8	374
Not stated	-	19	-	18	-	16	-	16
**Drinking risk** ^**b**^								
Lower-risk (≤4 AUDIT-C)	46.0	392	45.2	385	48.6	419	49.0	421
Higher-risk (≥5 AUDIT-C)	54.0	461	54.8	467	51.4	443	51.0	439
Not stated^c^	-	30	-	29	-	29	-	29
AUDIT-C average score (*SD*)	5.03 (*2.67*)		5.09 (*2.72*)		4.96 (*2.61*)		4.94 (*2.62*)	

Notes: All percentages (%) are valid, i.e. excluding missing data (*Don’t know/Prefer not to say*) on alcohol consumption variables;^a^ Based on the three Ulster counties that are in Ireland, not the six Ulster counties in Northern Ireland;^b^ Base = All current drinkers in each wave;^c^ Data missing on at least one of AUDIT-C question two (standard drinks consumed on a typical drinking occasion) or three (frequency of HED); SD = Standard Deviation

### Sample

A cross-sectional sample of adults (18+) in Ireland were recruited at each wave. The unweighted and weighted sample characteristics at wave one (*n* = 1,007) and wave two (*n* = 1,020) are reported in Table 1. YouGov, a market research company, carried out both waves through their RealTime Omnibus service.^41^ They recruited a sample intended to be representative of the demographic profile of the Irish population through email invitations to members of their online panel. Although online market research panels are non-probability, they are suitable for such research providing best practice guidelines are adhered to and caveats around generalisability stated.^42–44^ YouGov’s panel is also frequently used for health policy research.^45–47^ A cross-sectional survey weight was provided for each wave to enable descriptive data to be representative of the demographic profile of the adult Irish population.

### Measures

#### Demographics

YouGov provided information on age, gender, and region from data held about panel respondents (Table 1).

#### Awareness of alcohol marketing

Marketing awareness was assessed using self-reported prompted recall. Although this approach is only one of the myriad ways exposure can be measured,^11^^,^^48–50^ it has been frequently used to evaluate the impact of marketing controls using repeat survey designs.^29^^,^^30^ In both waves, participants were prompted with the statement ‘*Thinking about the last month how often, if at all, have you…*’ and presented with a list of 13 marketing activities (Table 2). At wave two, additional items were added for adverts on podcasts or audio streaming services and internet celebrities (e.g., social influencers) to capture emergent marketing activities.^51^ In both waves, frequency of awareness was reported on a six-point scale (*1 = ‘Everyday’* to *6=‘Not at all;* or ‘*Not sure if seen in the last month*’).

**Table 2:** Any self-reported awareness of each marketing activity in the past month and changes between survey waves

	Reported any awareness in the past month				
	W1	W2	Logistic regression (W1 = 0; W2 = 1)^f^		
Marketing activity^a^	%	%	*OR*_*Adj*_	95% CI	p
***Adverts for alcohol…***					
**1.** … in newspapers or magazines	72.6	63.7	0.67	0.55–0.83	<0.001
**2.** … on television (incl. prog. sponsorship)^b^	86.0	79.4	0.65	0.51–0.83	0.001
**3.** … on public transport/transport hubs (R)	64.5	54.6	0.66	0.53–0.81	<0.001
**4**. … on catch-up or streaming services	49.0	51.6	1.18	0.96–1.44	0.120
**5**. … on posters and billboards (R)	77.0	69.2	0.66	0.53–0.83	<0.001
**6**. … at the cinema (R)	37.1	27.0	0.58	0.46–0.73	<0.001
**7**. … on the radio	55.9	50.3	0.80	0.66–0.98	0.030
**8**. … on YouTube, Tumblr, Facebook, Snapchat, Instagram or other social media	57.0	55.3	0.95	0.76–1.18	0.638
**9**. … on podcast/audio streaming services^c^	-	38.1	-	-	-
**10**. Famous people in films, music videos, on TV or pictured in magazines with alcohol	81.7	75.6	0.70	0.55–0.89	0.003
**11**. Seen merchandise with an alcohol brand logo, such as clothing, glasses or other items	74.5	69.6	0.81	0.65–1.00	0.054
**12**. Sport or event sponsorship	86.0	75.0	0.47	0.37–0.61	<0.001
**13**. Special price offers	86.6	85.5	0.93	0.71–1.22	0.611
**14**. Competitions	56.6	49.6	0.75	0.61–0.92	0.006
**15.** Seen internet celebrities (e.g. YouTubers) talking about, or promoting, an alcohol brand^c^	-	44.8	-	-	-
**Awareness of any marketing activity** ^**d,e**^	94.1	93.8	0.95	0.65–1.37	0.767

Notes: (R) = Full or partial restrictions implemented on this activity between survey waves; W1 = October 2019 (pre-initial restrictions; pre-COVID-19 pandemic); W2 = October 2020 (post-initial restrictions and during COVID-19 pandemic); *OR_Adj_* = Adjusted Odds Ratio; 95% CI = 95% Confidence Interval. Percentages (%) are weighted. Logistic regressions unweighted as age, gender, and region included as covariates.

^a^Base = Those who provided a valid answer to each marketing activity in each wave (i.e., provided accurate estimate or said ‘*not at all’*; those saying ‘*not sure*’ excluded activity-by-activity).

^b^Phrase ‘including programme sponsorship’ only added for wave two.

^c^Marketing activity only measured in W2 so no between-wave comparison.

^d^Base = All participants in each wave (2019: *n* = 1,007; 2020: *n* = 1,020)

^e^Comparison based only on the 13 activities measured in both 2019 and 2020 waves.

^f^Omnibus test of model coefficients for all models was *p* < 0.001, except special prices offers (*p* = 0.002). Hosmer & Lemeshow *p* > 0.05 for all models, except competitions (*p* = 0.012).

Four variables were derived for marketing awareness. First, participants were binary coded based on whether they reported any past-month awareness for each activity (*Yes/No*; *Not sure* excluded) and past-month awareness of any activity (*Yes/No*). The variable examining past-month awareness of any activity only considered the 13 activities measured in both waves. Second, the self-reported frequency of awareness was converted into the estimated number of times marketing had been seen in a four-week period (one month); for example, ‘*Everyday*’ equalled 28 instances. Third, an aggregate awareness score was computed by summing frequencies for participants who provided a valid answer for all activities measured in both waves (wave one: *n* = 420; wave two: *n* = 476). This score ranged from 0 (saw no marketing) to 364 (every activity, everyday). Finally, the aggregate awareness score was divided into low, medium, and high categories using unweighted tertile splits. To ensure the categories were sensitive to the point of measurement, they were based on the cross-sectional boundaries, not a grand sample. In wave one the splits were: low = < 48 instances; medium = 49–129; high= > 130. In wave two they were: low = < 42; medium = 43–112; high= > 113.

### Alcohol consumption

Participants completed the Alcohol Use Disorders Identification Test-Concise (AUDIT-C), which measured frequency of consumption, standard drinks consumed on a typical drinking occasion (one standard drink = 10 grams of pure alcohol^52^), and frequency of HED (≥six standard drinks [≥60 g pure alcohol] on a single occasion). All participants who answered ‘*never*’ to the first item, frequency of consumption, were classified as ‘*non-drinkers*’ and did not complete the remaining items. The remainder were classified as ‘*current drinkers*’ and completed all items. Those who said ‘*Don’t know/Can’t recall’* for frequency of consumption were treated as missing for drinking status.

Two variables were derived from the AUDIT-C to capture the riskier consumption patterns the Act aims to address. First, current drinkers were divided into those who engaged in HED at least monthly versus those who did so less often or never (unspecified responses were excluded); at least monthly HED is consistent with the past 30-day timeframes suggested by the WHO.^53^ Second, a composite score was computed for current drinkers who provided a valid answer to all AUDIT-C items (i.e., did not say ‘*Don’t know/Prefer not to say*’ for the second and third items). AUDIT-C scores can range 0–12 and, consistent with concurrent approaches in Ireland, ≥ 5 was classified as higher-risk consumption.^54^^,^^55^

### Analysis

Data were analysed using SPSS version 23 (Chicago, IL). Analyses are unweighted unless stated. Weighted Chi-squares examined whether the proportion of current drinkers, current drinkers engaging in at least monthly HED, and current drinkers consuming at higher-risk varied by wave. Among current drinkers, a weighted independent samples *t*-test examined differences in AUDIT-C score between waves.

In each wave, weighted frequencies examined the proportion of participants aware of each marketing activity in the past month and the proportion aware of any activity. Logistic regressions examined whether awareness varied by wave, overall and for each activity. In each model, the dependent variable was whether any awareness was reported and the key independent variable was wave. Each model controlled for age, gender, and region. Frequencies examined estimated frequency of seeing marketing in the past month, for both individual activities and the aggregate estimate. Mann Whitney tests examined differences by wave, overall and for each activity.

Logistic regressions were computed among current drinkers with level of risk (*higher-risk vs. lower*) and HED (*at least monthly vs. less often/never*) the dependent variables. Past-month self-reported awareness (*low/medium/high/not stated*) was the key independent variable. Both models controlled for age, gender, region, and wave. In each model, a final block examined whether there was an interaction between wave and self-reported awareness. The reference categories and contrast functions for covariates are reported in the results.

### Ethics

University of Stirling’s General University Ethics Panel (wave one: GUEP756; wave two: GUEP[19/20]963).

## Results

### Changes in alcohol consumption

Weighted Chi-squares found no difference between waves for the proportion of participants who were current drinkers (*χ^2^* = 0.11, *p* = 0.738), the proportion of current drinkers who reported at least monthly HED (*χ^2^* = 0.15, *p* = 0.702), and the proportion of current drinkers categorised as higher-risk (*χ^2^* = 2.44, *p* = 0.119) (Table 1). Among current drinkers, a weighted independent samples *t*-test found no difference between waves in AUDIT-C score (*t* = 1.17, *p* = 0.241).

### Changes in where alcohol marketing was seen

In 2019, 94.1% of participants recalled seeing at least one instance of alcohol marketing in the past month, while 93.8% did in 2020 (Table 2). A logistic regression, controlling for age, gender, and region, found the likelihood of recalling any awareness in the past month did not vary by wave (*OR_Adj_* = 0.95, *p* = 0.767). For 9/13 activities measured at both waves, the likelihood of recalling any past-month awareness was lower in 2020 than in 2019 (*OR_Adj_* range: 0.47 to 0.80; *p* range: < 0.001 to 0.030) (Table 2). These decreases included activities at least partly restricted in November 2019; public transport (*OR_Adj_* = 0.66, *p* < 0.001), posters and billboards (*OR_Adj_* = 0.66, *p* < 0.001), and cinema (*OR_Adj_* = 0.58, *p* < 0.001). No change was observed for catch-up or streaming services (*p* = 0.120), social media (*p* = 0.638), special price offers (*p* = 0.611), and branded merchandise (*p* = 0.054).

**Table 3:** Self-reported frequency of awareness for each marketing activity in the past month and changes between survey waves

	Estimated frequency of awareness in the past month					
	W1		W2		Mann Whitney	
Marketing activity^a^	*Mdn*	*IQR*	*Mdn*	*IQR*	*p*	*r*
***Adverts for alcohol…***						
**1** … in newspapers or magazines	2	0–14	2	0–6	<0.005	−0.07
**2** … on television (incl. prog. sponsorship)^b^	6	2–22	6	2–14	<0.001	−0.11
**3** … on public transport/transport hubs (R)	2	0–14	2	0–6	<0.001	−0.10
**4**. … on catch-up or streaming services	0	0–6	2	0–6	0.126	0.04
**5**. … on posters and billboards (R)	6	2–14	6	0–14	<0.001	−0.10
**6**. … at the cinema (R)	0	0–2	0	0–2	0.001	−0.08
**7**. … on the radio	2	0–6	2	0–6	0.064	−0.04
**8**. … on YouTube, Tumblr, Facebook, Snapchat, Instagram or other social media	2	0–14	2	0–14	0.431	−0.02
**9**. … on podcast/audio streaming services^c^	-	-	0	0–6	-	-
**10**. Famous people in films, music videos, on TV or pictured in magazines with alcohol	6	2–14	6	2–14	0.024	−0.05
**11**. Seen merchandise with an alcohol brand logo, such as clothing, glasses or other items	2	0–14	2	0–14	0.132	−0.04
**12**. Sport or event sponsorship	6	2–14	6	2–14	<0.001	−0.11
**13**. Special price offers	6	2–14	6	2–14	0.040	−0.05
**14**. Competitions	2	0–6	2	0–6	0.021	−0.06
**15.** Seen internet celebrities (e.g. YouTubers) talking about, or promoting, an alcohol brand^c^	-	-	0	0–6	-	-
**Aggregate awareness across activities** ^**d,e**^	85	34–156	74	24.5–146	0.047	−0.07

Notes: (R) = Full or partial restrictions implemented on this activity between survey waves; W1 = October 2019 (pre-initial restrictions; pre-COVID-19 pandemic); W2 = October 2020 (post-initial restrictions and during COVID-19 pandemic); *Mdn* = Median; *IQR* = Inter Quartile Range. Both descriptive data and tests are unweighted as tests are non-parametric.

^a^Base = Those who provided a valid answer to each marketing activity in each wave (i.e., provided accurate estimate or said ‘*not at all’*; those saying ‘*not sure*’ excluded activity-by-activity).

^b^Phrase ‘including programme sponsorship’ only added for wave two.

^c^Marketing activity only measured in W2 and, therefore, no between-wave comparison.

^d^Comparison based only on the 13 activities measured in both the 2019 and 2020 waves.

^e^Base = All participants who provided valid answer to all activities measured in both waves (2019: *n* = 420; 2020: *n* = 476).

### Changes in how often alcohol marketing was seen

There was a decrease in aggregate past-month awareness reported across activities between 2019 (*Mdn* = 85 instances, *IQR* = 34–156) and 2020 (*Mdn* = 74 instances, *IQR* = 24.5–146). A Mann Whitney test showed this change to be significant, albeit the effect size was small (*p* = 0.047, *r* = −0.07). For 9/13 activities measured at both waves, Mann–Whitney tests showed that awareness was lower in 2020 compared to 2019 (*p* range: < 0.001 to 0.040) (Table 3), albeit the effect sizes were small (*r* range: −0.05 to −0.11). These decreases included activities at least partly restricted from November 2019; public transport (*p* < 0.001, *r* = −0.10), posters and billboards (*p* < 0.001, *r* = −0.10), and cinema (*p* = 0.001, *r* = −0.08). No change was observed for catch-up or streaming services (*p* = 0.126), social media (*p* = 0.431), radio (*p* = 0.064), and branded merchandise (*p* = 0.132).

### Association between alcohol marketing and consumption

Across waves, a logistic regression showed that self-reported marketing awareness was associated with at least monthly HED among current drinkers (*Wald χ^2^* = 33.28, *p* < 0.001) (Table 4). Specifically, current drinkers who reported medium (*OR_Adj_* = 1.83, *p* = 0.001) or high past-month awareness (*OR_Adj_* = 2.55, *p* < 0.001) were more likely to report at least monthly HED than current drinkers reporting low awareness. A further block, testing the interaction between wave and awareness, found that the association between marketing and at least monthly HED did not vary over time (*Wald χ^2^* = 1.66, *p* = 0.645).

**Table 4:** Binary logistic regressions examining: (1) the associations between self-reported past-month alcohol marketing awareness and at least monthly high-episodic drinking (HED) and higher-risk consumption among current drinkers and (2) the interaction between marketing awareness and survey wave

	At least monthly HED^a,b^				Higher-risk drinking^c,d^			
Variables	n	*OR*_*Adj*_	*95% CI*	p	n	*OR*_*Adj*_	*95% CI*	p
**Age group**								
18–24 years	187	REF	-	0.015	182	REF	-	0.583
25–34 years (*vs. younger*)	336	0.76	0.52–1.10	0.144	332	1.01	0.69–1.48	0.955
35–44 years (*vs. younger*)	390	0.78	0.59–1.03	0.079	386	0.84	0.63–1.12	0.243
45–54 years (*vs. younger*)	353	0.98	0.75–1.27	0.863	347	0.87	0.66–1.14	0.303
≥55 years (*vs. younger*)	473	0.70	0.55–0.88	0.002	468	0.90	0.71–1.13	0.359
**Gender**								
Female	906	REF	-	-	893	REF	-	-
Male	833	2.27	1.85–2.77	<0.001	822	2.71	2.21–3.32	<0.001
**Region/County**								
Dublin	561	REF	-	0.125	557	REF	-	0.303
Rest of Leinster (*vs. Dublin*)	442	0.81	0.62–1.06	0.121	436	0.85	0.65–1.11	0.226
Munster (*vs. Dublin*)	427	0.92	0.70–1.19	0.515	419	0.94	0.72–1.23	0.641
Connaught & part of Ulster (*vs. Dublin*)	309	0.71	0.53–0.96	0.027	303	0.77	0.57–1.03	0.078
**Self-reported marketing awareness**								
Low awareness	259	REF	-	<0.001	254	REF	-	<0.001
Medium awareness (*vs. Low*)	273	1.83	1.27–2.64	0.001	272	1.67	1.16–2.40	0.006
High awareness (*vs. Low*)	268	2.55	1.75–3.72	<0.001	266	2.28	1.55–3.34	<0.001
Not stated (*vs. Low*)	939	1.25	0.93–1.69	0.140	923	1.16	0.86–1.55	0.325
**Survey wave**								
Wave One (Pre-implementation, Pre-COVID-19)	864	REF	-	-	853	REF	-	-
Wave Two (Post-implementation, COVID-19)	875	0.95	0.78–1.16	0.613	862	0.84	0.68–1.02	0.079
Survey wave^*^Marketing awareness (separate block)	*Wald χ^2^* (3) =1.66, *p* = 0.645				*Wald χ^2^* (3) =6.68, *p* = 0.083			

Notes: Base = All current drinkers in each wave (*n* = 1,774); *OR*_*Adj*_ = Adj. Odds Ratio; 95% CI = 95% Confidence Interval; Data are unweighted. DVs = At least monthly Heavy Episodic Drinking (HED vs. Less often/Never); Higher-risk consumption (vs. Lower-risk consumption). Model summaries for *main effect block* (i.e. not including wave^*^marketing interaction, which was entered in separate block after main effects).

^a^Test of coefficients, *χ^2^*(12) = 145.33, *p* < 0.001; Hosmer & Lemeshow, *χ^2^*(8) = 9.31, *p* = 0.317, Nagelkerke *R^2^* = 0.108.

^b^Cases excluded due to missing data on HED item (*n* = 35).

^c^Test of coefficients, *χ^2^*(12) = 157.67, *p* < 0.001; Hosmer & Lemeshow, *χ^2^*(8) = 7.07, *p* = 0.529, Nagelkerke *R^2^* = 0.117.

^d^Cases excluded due to missing AUDIT-C score (*n* = 59).

Across waves, a logistic regression showed that self-reported awareness was associated with higher-risk drinking among current drinkers (*Wald χ^2^* = 25.35, *p* < 0.001) (Table 4). Specifically, current drinkers who reported medium (*OR_Adj_* = 1.67, *p* = 0.006) or high past-month awareness (*OR_Adj_* = 2.28, *p* < 0.001) were more likely to report higher-risk drinking than those reporting low awareness. A further block, testing the interaction between survey wave and awareness, found that the association between marketing and higher-risk drinking did not vary over time (*Wald χ^2^* = 6.68, *p* = 0.083).

## Discussion

### Main findings of this study

These repeat cross-sectional surveys show that one year after initial implementation of Ireland’s marketing controls, and during the COVID-19 pandemic, self-reported awareness of alcohol marketing decreased. For 9/13 activities measured at both waves, there were decreases in the proportion who recalled any past-month awareness and estimated frequency of awareness. Nevertheless, awareness remained high at wave two, with around nine-out-of-ten participants recalling at least one form of alcohol marketing, at least half reporting seeing 74 or more instances in the past month, and at least half aware of most marketing activities. Among current drinkers, there was an association between marketing awareness and higher-risk consumption patterns across waves.

### What is already known on this topic

Despite research consistently reporting a causal link between alcohol marketing and consumption,^10–13^ and harm reduction organisations recommending statutory restrictions,^20^^,^^56^^,^^57^ there remain at least three important gaps. First, although some countries already have statutory controls,^21–26^ there has been little examination of pre-and-post-effect on marketing awareness and consumption among consumers.^28^ Second, while previous research of alcohol industry activity has suggested that marketing may encourage risky consumption among adults,^34^ most studies have focused on young people.^10–12^ Finally, although some studies have examined how alcohol marketing has changed with the COVID-19 pandemic,^35^^,^^36^ there is little evidence about how marketing awareness changed among consumers due to restrictions on social interaction.

### What this study adds

To our knowledge, this is the first study to examine awareness of a comprehensive array of alcohol marketing activities among adults before, and after, the implementation of statutory controls. Awareness decreased for marketing activities subject to new restrictions from November 2019, overall and by frequency. This is consistent with evaluations of statutory marketing controls for other fast-moving consumer goods.^29^^,^^30^

It is plausible, however, that COVID-19 restrictions may have also contributed to reductions in awareness for the marketing activities subject to the initial controls. Community mobility data, for example, reports that footfall on public transport in Ireland was approximately two-fifths lower when the wave two data were collected compared to pre-pandemic levels,^58^ and cinemas were closed or had reduced capacity.^59^^,^^60^ Reduced footfall in urban spaces, due to restrictions on retail or recreational outlets and the requirements for social distancing, also reportedly led to reductions in advertising spend and intensity for out-of-home media, which may have removed some opportunities for exposure.^61^ Nevertheless, the wave two data were collected while some degree of exposure to these activities was possible, unlike other stages of the pandemic when national lockdowns precipitated closure of public spaces and mandated stay-at-home advice. We therefore recommend a precautionary interpretation. It is plausible both the Act’s controls and the COVID-19 pandemic contributed to decreases in awareness, but longer-term evaluation is required to determine their relative contribution. We will be conducting follow-up data collection in October 2021.

This is also the first study to examine how the COVID-19 pandemic influenced alcohol marketing awareness. Data show the pandemic led to reductions in where, and how often, consumers recalled seeing marketing activities that were not subject to new legislative restrictions. For example, cancellation of mass participation events and requirements for sport to have limited (or no) spectators led to reductions in awareness of sponsorship. There were activities, however, in which awareness did not reduce, such as adverts on catch-up and streaming services or social media. This is logical given such marketing can be served in-home. Nevertheless, despite some declines, over nine-out-of-ten participants recalled seeing at least one form of marketing at wave two and at least half recalled some awareness for 11/15 activities measured at wave two. That alcohol marketing was still able to reach consumers during the pandemic is consistent with research analysing marketing activity during this period.^35^^,^^36^ This finding may also, at least partly, help to explain the sustained levels of alcohol consumption during the pandemic, a trend reported in this study and elsewhere.^4^^,^^5^

Irrespective of the legislative and COVID-19 context, this study contributes new evidence about adults’ experience of alcohol marketing. The data show that, at both waves, participants recalled seeing marketing through a variety of activities, at least half were estimated to have seen marketing 2–3 times per-day or more in the past month, and increased awareness was associated with at least monthly HED and higher-risk drinking. These trends are consistent with research that suggests adults are important targets for alcohol marketing,^19^^,^^34^ and support Ireland’s approach for introducing measures that reduce population-level exposure to marketing (e.g., public transport advertising) as well as targeted restrictions among young people.

### Limitations of this study

Both waves come from a non-probabilistic online panel which, albeit an established approach in health policy research,^45–47^ limits external generalisability. We also only consider adults as an aggregate sample. Findings may differ by demography and other vulnerabilities within the adult population (e.g., dependent drinkers) or among consumers below the minimum legal purchase age. While the repeat cross-sectional design meant no attrition, it cannot show a causal link between marketing and consumption. Furthermore, although research suggests that the influence of marketing is cumulative across activities,^62^ use of an aggregate score when examining associations with consumption removes the sensitivity that some activities and brands are likely to have a stronger association than others. The total awareness score was also only based on the activities measured in both waves, and therefore likely underestimates overall awareness and the associations with consumption.

The self-reported data are subject to recall errors for alcohol use and marketing awareness. For example, around half of participants reported seeing advertising on public transport at wave two, despite this activity being prohibited. Such reporting may relate to genuine recall errors, recalling marketing near to but not part of public transport (e.g., outdoor advertising or licenced premises close to transport stops), erroneous conflation with other activities (e.g., seeing print advertising or branded merchandise on public transport), or possible regulatory infringements. For the latter, research examining compliance would be beneficial, as has been the case in other countries where alcohol marketing has been restricted.^25^^,^^26^ Future research using methods applied elsewhere in alcohol marketing literature, for example ecological momentary assessments^49^ and wearable cameras,^50^ would also provide granular understanding of the marketing activities seen (e.g., test hypotheses for continued public transport exposure) and capture instances of marketing not consciously recognised or recalled by participants.

Due to cost, we were unable to capture changes to personal circumstances resulting from the pandemic. Doing so would have enabled examination of specific reasons for awareness decreases during COVID-19, for example ‘cocooning’ or increased caring responsibilities limiting exposure opportunities, rather than post-hoc hypothesising using extant literature. We also did not have access to data concerning alcohol marketing activity in Ireland, for example advertising expenditure, volume, and placement. Such information would have helped to contextualise how variations in consumer awareness corroborated longer-term shifts in alcohol marketing strategy (e.g., from traditional media to convergent marketing activities) or voluntary changes in marketing practice in anticipation of the legislation.

## Supplementary Material

S1_-_What_is_in_the_CV19_levels_in_Ireland_fdab353

## Data Availability

Data available upon reasonable request from the corresponding authors.

## Notes

**Nathan Critchlow,** SSA Academic Fellow

**Crawford Moodie,** Senior Research Fellow
