# Supplementary material for: Awareness of alcohol marketing one year after initial implementation of Ireland’s Public Health (Alcohol) Act and during the COVID-19 pandemic
Source: J Public Health (Oxf). 2021 Oct 9;44(4):e537–47. doi: 10.1093/pubmed/fdab353 (PMC9715295; doi:10.1093/pubmed/fdab353)
Supplement: S1_-_What_is_in_the_CV19_levels_in_Ireland_fdab353 [file s1_-_what_is_in_the_cv19_levels_in_ireland_fdab353.pdf]

National Framework for living with COVID-19

Coronavirus COVID-19 National Programme

Very low rate of COVID-19 infections Low R-Number → Very high rate of COVID-19 infections High R-Number

	Level 1	Level 2	Level 3	Level 4	Level 5
**Social & Family Gatherings**	Max: **10** from **3** other households	Max: **6** from **3** other households	Max: **6** from **1** other household	No visitors	No visitors
**Weddings**	Max: **100**	Max: **50**	Max: **25**	Max: **6**	Max: **6**
**Indoor Events**	Depending on venue size **100** | **200**	Depending on venue size **50** | **100**	No organised events	No organised events	No organised events
**Outdoor Events**	Depending on venue size **200** | **500**	Depending on venue size **100** | **200**	Gatherings of up to **15**	Gatherings of up to **15**	
**Sports Training**	Normal training with protective measures	**Indoors** Pods of **6**; **Outdoors** Pods of **15**	**1** Individual only; Pods of **15** Non-contact	**1** Individual only; Pods of **15** Non-contact	Individual training only
**Matches & Events**	**100** indoor, **200** outdoor, **500** stadia	**50** indoor, **100** outdoor, **200** stadia	Except specific exemptions	Except specific exemptions	No events
**Gyms, Pools & Leisure Centres**	**Open** with protective measures	**Open** with protective measures	**Individual** training only	Closed	Closed
**Religious Services**	**50** (or sub-groups of 50)	**50** (or sub-groups of 50)	Services move online; **Funerals 25**	Services move online; **Funerals 25**	Services move online; **Funerals 10**
**Bars serving food, Cafés & Restaurants**	**Open** with protective measures	**Open** Groups of **6** from up to **3** households	Range of restrictions up to and including no indoor dining	**Outdoor** dining only Max: **15** Takeaway/Delivery	**Takeaway/Delivery** only
**Wet Pubs**	**Open** with protective measures	**Open** Groups of **6** from up to **3** households	Range of restrictions up to and including no indoor service*	**Outdoor** only Max: **15** persons Takeaway/Delivery	**Takeaway/Delivery** only
**Hotels, Guesthouses B&Bs**	**Open** with protective measures	**Open** with protective measures	Services limited to **Residents** only	**Existing Guests & Essential Purposes** only	**Essential Purposes** only
**Retail & Services** (Mandatory face coverings)	**Open** with protective measures	**Open** with protective measures	**Open** with protective measures	Primarily outdoor **Essential retail/services**	**Essential Retail** only
**Indoor Cultural Venues**	**Open** with protective measures	**Open** with protective measures	**Venues closed** Libraries available for e-services/call & collect	**Venues closed** Online services available	**Venues closed** Online services available
**Workplace** (Work from home if possible)	**Staggered attendance**	**Essential** Meetings, Induction & Training only	**Attend only if absolutely necessary**	**Essential & other designated workers** only	**Essential workers** only
**Domestic Travel**	**No restrictions**	**No restrictions**	**Stay in your county** except for work, education & essential purposes	**Stay in your county** Except for essential work, education & essential purposes	**Stay at home** Exercise within 5km
**Public Transport** (Cycle or walk if possible; Mandatory face coverings)	**Off-peak hours**	**50%** Capacity; **Peak hours reserved** for essential workers and purposes	**50%** Capacity; **Only when necessary**	**25%** Capacity; **Avoid public transport**	**25%** Capacity; **Avoid public transport**
**Schools & Childcare**	**Open** with protective measures	**Open** with protective measures	**Open** with protective measures	**Open** with protective measures	Recommendations based on situation and evidence at time
**Adult & Higher Education**	**Open** with protective measures	**Open** with protective measures	**Open** Escalate all appropriate protective measures / limit congregation	**Move online** On-site attendance only when essential	Recommendations based on situation and evidence at time
**Care Homes**	**Open** with protective measures	**Open** with enhanced protective measures	**Visiting suspended**, aside from critical and compassionate circumstances		
**Over 70s & Medically Vulnerable**	Exercise judgement regarding engaging with others and in activities outside home.				

*Pubs currently remain closed in Dublin

More detailed information is available at **gov.ie/COVID19**

Rialtas na hÉireann
Government of Ireland
